# Pleiotropic Effects of Eugenol: The Good, the Bad, and the Unknown

**DOI:** 10.1155/2021/3165159

**Published:** 2021-03-02

**Authors:** Oana M. Aburel, Ioana Z. Pavel, Maria D. Dănilă, Theia Lelcu, Alexandra Roi, Rodica Lighezan, Danina M. Muntean, Laura C. Rusu

**Affiliations:** ^1^Department of Functional Sciences-Pathophysiology, Faculty of Medicine, “Victor Babes” University of Medicine and Pharmacy, Eftimie Murgu Sq. No. 2, 300041 Timișoara, Romania; ^2^Center for Translational Research and Systems Medicine, Faculty of Medicine, “Victor Babeș” University of Medicine and Pharmacy, Eftimie Murgu Sq. No. 2, 300041 Timișoara, Romania; ^3^Department II-Pharmacognosy, Faculty of Pharmacy, “Victor Babeș” University of Medicine and Pharmacy, Eftimie Murgu Sq. No. 2, 300041 Timișoara, Romania; ^4^Pharmaco-Toxicological Evaluations Research Center, Faculty of Pharmacy, “Victor Babeș” University of Medicine and Pharmacy, Eftimie Murgu Sq. No. 2, 300041 Timișoara, Romania; ^5^Department-Oral Pathology, Faculty of Dentistry, “Victor Babeș” University of Medicine and Pharmacy, Eftimie Murgu Sq. No. 2, 300041 Timișoara, Romania; ^6^Multidisciplinary Center for Research, Evaluation, Diagnosis and Therapies in Oral Medicine, Faculty of Dentistry, “Victor Babeș” University of Medicine and Pharmacy, Eftimie Murgu Sq. No. 2, 300041 Timișoara, Romania; ^7^Department of Infectious Diseases-Parasitology, Faculty of Medicine, “Victor Babes” University of Medicine and Pharmacy, Eftimie Murgu Sq. No. 2, 300041 Timișoara, Romania; ^8^Center for Diagnostic and Study of Parasitic Diseases, Faculty of Medicine, “Victor Babeș” University of Medicine and Pharmacy, Eftimie Murgu Sq. No. 2, 300041 Timișoara, Romania

## Abstract

Phytocompounds and medicinal herbs were used in traditional ancient medicine and are nowadays increasingly screened in both experimental and clinical settings due to their beneficial effects in several major pathologies. Similar to the drug industry, phytotherapy is interested in using nanobased delivery systems to view the identification and characterization of the cellular and molecular therapeutic targets of plant components. Eugenol, the major phenolic constituent of clove essential oil, is a particularly versatile phytochemical with a vast range of therapeutic properties, among which the anti-inflammatory, antioxidant, and anticarcinogenic effects have been systematically addressed. In the past decade, with the emerging understanding of the role of mitochondria as critical organelles in the pathophysiology of noncommunicable diseases, research regarding the role of phytochemicals as modulators of bioenergetics and metabolism is on a rise. Here, we present a brief overview of the major pharmacological properties of eugenol, with special emphasis on its applications in dental medicine, and provide preliminary data regarding its effects, alone, and included in polyurethane nanostructures, on mitochondrial bioenergetics, and glycolysis in human HaCaT keratinocytes.

## 1. Introduction

Eugenol (4-allyl-2-methoxyphenol) is the major volatile, biologically active component of clove oil, classically obtained from the dried flower buds of *Eugenia caryophyllata* Thunb. (Myrtaceae) [1]. This phytochemical has emerged from ancient times as a versatile molecule with a plethora of applications in drug, food and cosmetic industries, and agriculture [2]. In medicine, eugenol is best known for its original use in dentistry as cavity filling cement with local antiseptic and analgesic effects [3, 4]. However, the compound has been systematically investigated for numerous other pharmacological activities, such as anti-infective (antimicrobial, antihelmintic, antiviral, antifungal, antiparasitic, and insecticidal) [5, 6], anti-inflammatory, antioxidant [7, 8], and anticarcinogenic, when administered alone or in synergistic association with conventional therapies [9–11].

Modulation of multiple intracellular signaling pathways is the hallmark of most phytochemicals, and a tremendous amount of research is currently aimed at providing their thorough characterization. This is particularly true for their counteracting effects against oxidative stress and low-grade chronic inflammation, the major interconnected pathomechanisms of noncommunicable diseases (cardiometabolic, renal, liver pathologies, and cancer), and ageing [12]. Eugenol has elicited dose-dependent radical scavenging and anti-inflammatory activities in various *in vitro* experiments and animal models of chronic diseases [13], as well as antiproliferative and cytotoxic effects on several cancer cell lines and tumors [14, 15].

Phytochemicals present the advantages of low toxicity and high tolerability but there is an unmet need to both prevent their early metabolization and direct them towards the subcellular specific domains of action. Nowadays, an increasing amount of research is aimed at enhancing bioavailability and providing targeted delivery of natural compounds (recently reviewed in refs. [16, 17]). In the past decade, several natural product-based nanoformulations using polyurethane structures have been prepared, yielding promising results [18–23].

After oral administration in humans, eugenol is rapidly absorbed, metabolized, and almost completely excreted into urine as sulphate or glucuronide conjugates [24]. To overcome these disadvantages, a previous study reported the encapsulation of eugenol in polyurethane nanostructures with good thermal stability and encapsulation efficiency that can be further used for *in vitro* and *in vivo* testing [25].

Despite the fact that prolongation of the circulating lifetime and/or cellular entry may be facilitated by nanocarriers, the effects of these particles on various organelles require a thorough characterization. This is particularly true for mitochondria, organelles that are currently viewed as integrative hubs for energetics, redox control, and in/out signaling of almost all cells; indeed, it is mitochondrial dysfunction that triggers oxidative stress, potentiates inflammation in the setting of chronic pathologies [26], and influences all steps of oncogenesis, including cancer progression [27, 28].

The present paper is double-aimed (i) to provide a brief overview of eugenol pleiotropic cellular effects with a particular emphasis on its controversial role in dental medicine and (ii) to present preliminary data regarding the effects of eugenol, alone, and in polyurethane nanoformulations, on mitochondrial bioenergetics, and glycolysis in HaCaT human keratinocytes.

## 2. Overview of the Eugenol Use in Dental Medicine

Eugenol belongs to the phenol propanoid class (C_10_H_12_O_2_) and is, probably, the compound with the longest history of use in dental medicine in association with other materials, the most popular being a zinc oxide-eugenol (ZOE) paste. ZOE is obtained by mixing the zinc oxide powder with the liquid eugenol resulting in a zinc eugenolate chelate matrix. Owing to advantages such as low cost, good sealing, and easy handling, ZOE formulations have been widely used since the beginning of the last century as temporary restorative or impression materials, cements, bases, and liners and have also been incorporated in various endodontic sealers [29–31].

After filling a dentinal cavity with ZOE temporary cements, low amounts of eugenol slowly diffuse through the dentin tubules and exert anti-inflammatory, immune-modulatory [32], antinociceptive effects on the dental pulp, and sensitive teeth [29, 33, 34] together with antibacterial and anticariogenic activities [35, 36]. The anti-inflammatory effect of eugenol has been widely reported by several studies, being ascribed to the following mechanisms: (i) inhibition of the synthesis of inflammatory mediators by interference with the arachidonic acid metabolism [37], particularly via the cyclooxygenase pathway (decreased prostaglandins and thromboxanes) and less via the lipooxygenase pathway (decreased leukotrienes) [38–40], (ii) inhibition of neutrophil chemotaxis and decreased superoxide generation [41], and (iii) reduction of pain via inhibition of the periapical/intradental nerve activity [42, 43]. More recently, the beneficial role of eugenol-based paste on preventing alveolar osteitis and promoting superior wound healing was reported in a study that included 270 patients having the third molars extracted [44].

At variance to their protective effects, ZOE-based materials were also reported to elicit local cytotoxicity, in particular pulpal chronic inflammation, degeneration, and even necrosis either when placed in direct contact with vital tissues or via diffusion across dentinal tubules. Among the presumed mechanisms, an increase in cell membrane permeability/injury (due to their lipophilicity), alteration of ionic homeostasis, oxidation by peroxidases (with subsequent formation of cytotoxic metabolites), and generation of reactive oxygen species (ROS) was mostly reported [42, 45].

There is a huge amount of research demonstrating the cytotoxicity of various ZOE cements on human primary/permanent cell lines and animal cell lines/models. Several conclusions can be drawn from these studies. First, the results of the cytotoxicity studies in animal-based cell models are different from the ones obtained in human cells, even for the same tested material. Thus, the Chinese hamster lung fibroblasts [46] or mouse fibroblasts [47, 48] are more sensitive to eugenol's toxic effects as compared to primary or immortalized human cell lines; accordingly, human cells should be used for the clinical relevance of these studies. Second, all ZOE-based root canal sealers dissolve when exposed to an aqueous environment for extended periods and may cause mild to severe cytotoxic reactions [49] with the highest toxic effect being recorded for the freshly mixed material [50]; thus, the time-dependent evolution of cytotoxicity should be equally addressed. Third, all sealing materials will trigger periapical inflammation when present in the apical tissues; therefore, confining the filling to the root canal (i.e., avoiding overfilling) is critical for preventing/reducing chronic inflammation [51]. In this regard, Hong et al. deliberately overfilled root canals of monkey incisors with two ZOE-based sealers and reported mild to severe irritation of the periapical tissues that persisted over the 6-month period of experimental follow-up [52].

Last but not least, an important yet rather less addressed issue in the literature, is the dose titration. Jeng et al. investigated the dose-dependency of cytotoxicity and reported that eugenol was toxic to primary oral mucosal fibroblasts in high concentrations (≥3 mmol/L), and cell death was associated with intracellular depletion of glutathione and ATP, respectively. At variance, a protective effect was described at lower concentrations (<1 mmol/L) presumably via the inhibition of xanthine oxidase activity and lipid peroxidation [53]. Comparable results with respect to total cell death were obtained when human diploid fibroblasts were incubated with high doses of eugenol (4 mM) [54]. Cytotoxicity of eugenol against normal human pulp fibroblasts was also demonstrated in terms of reduction of cell growth/survival and impairment of reparative processes, such as synthesis collagen and expression bone sialoprotein [55].

The group of Sagakami reported that eugenol elicited indiscriminate toxicity towards both normal human oral cells (cultured pulp cells, periodontal ligament fibroblasts, and gingival fibroblasts) and oral squamous cell carcinoma cell lines; specifically, eugenol induced rapid (after 4 h of incubation) nonapoptotic cell death with very low tumor specificity (IC 50 for normal cells was very close to the one for tumor cells) as compared to classic chemoterapeutic drugs [56]. These authors also reported a hormetic effect in cultured periodontal ligament fibroblasts (but interestingly, not in gingival fibroblasts) with an anti-inflammatory activity at lower doses that was lost when eugenol was applied in a higher dose [57]. Of note, a similar hormetic response (antioxidant at low doses, no effect, or prooxidant at high doses) was previously reported in the literature for another natural polyphenol, resveratrol [58].

Cytotoxic effects for ZOE and eugenol were reported not only for primary human oral cells but also towards immortalized human cells (dental pulp stem cells and oral keratinocytes), albeit in the latter case, zinc (and not eugenol) was considered to be responsible for most of the cytotoxicity [32, 56, 59]. Moreover, despite early ZOE toxicity, it was eugenol that downregulated the expression of the mRNA genes responsible for the synthesis of proinflammatory cytokines (IL-1, IL-6, and IL-8) in inflamed human dental pulp stem cells (but not in mouse bone marrow monocytes) [32].

A word of caution is in order in pediatric dentistry regarding eugenol genotoxicity. Escobar-Garcia et al. reported DNA damage in human pulp fibroblasts from primary teeth, when eugenol was applied in the lowest concentrations (0.06–5.1 *μ*M), an effect that, paradoxically, disappeared at higher concentrations (320 to 818 *μ*M) [60]. More recently, the same group reported that eugenol in low concentration (13 *μ*M) elicited an anti-inflammatory effect on cultured dental pulp fibroblasts exposed to lipopolysaccharide (LPS) that consisted in the inhibition of the gene expression of TNF-*α* (but not of IL-1*β*) and of the NF-*κ*B signaling pathway; unexpectedly, a proinflammatory effect was found for eugenol in non-LPS-exposed fibroblasts (i.e., in the absence of the induced inflammation) [61]. In a recent elegant study, Jeanneau et al. confirmed the anti-inflammatory properties of eugenol when applied alone on LPS-stimulated human periodontal fibroblasts assessed by its ability to inhibit the secretion of proinflammatory cytokines, IL-6 and TNF-*α*; however, the effects were not recapitulated when a ZOE cement was used. Moreover, neither eugenol alone nor the cement-based eugenol could decrease monocyte adhesion and migration as compared to a hydrocortisone-based cement. The authors concluded that the hydrocortisone (but not eugenol)-containing root sealers are able to modulate the initial steps of inflammation [62].

In isolated cases, eugenol was demonstrated to act as a contact allergen capable to trigger allergic responses, most frequently, by delayed hypersensitivity reactions (contact stomatitis), and rarely by type I hypersensitivity reactions (contact urticaria or even anaphylactic shock) [63–66].

Other disadvantages of eugenol/ZOE were published, such as inhibition of the polymerization of methacrylate monomers and resins, low mechanical strength, and limited durability (degradation occurs through hydrolysis) that might cause secondary fractures and reduction of the bond strength of posts luted to root canals [59, 67–70].

However, there is no general consensus in the literature regarding the “ugly” side of eugenol. Accordingly, in the past decade, several groups reported that ZOE is a suitable base material for composite resin restoration that did not affect (or even positively impacted) the composite polymerization measured by their microhardness [29, 71] and the bond strength [72]. Moreover, recent systematic reviews were not able to show evidence for the superiority of one sealing material over another with respect to biocompatibility and fracture resistance of endodontically treated teeth; also, only moderate evidence for the lack of a reinforcing effect for ZOE-based sealers was reported [73, 74]. The beneficial vs. deleterious effects of eugenol and ZOE are summarized in Table 1.

At variance from the conflicting results regarding the indications and contraindications of eugenol in dental medicine, there is a relative consensus in the literature on its beneficial effects in the setting of inflammation and cancer in both cell lines and animal models, as briefly described in the following subchapters.

## 3. Protective Cellular Effects of Eugenol: A Bird's Eye View

The link between inflammation and cancer was firstly proposed by the visionary German pathologist and anthropologist Rudolf Virchow, and the importance of preventing and/or reversing inflammation for the cancer control is nowadays widely recognized [75]. Eugenol exerts protective anti-inflammatory, antioxidant, and anticarcinogenic effects, as shown by several studies described below and summarized in Table 2.

### 3.1. Anti-Inflammatory and Antioxidant Activities of Eugenol

Inflammation is the natural response of our body against a variety of aggressors (physical or chemical agents, pathogens, injured cells, immune complexes, etc.) that exerts protective effects in the acute phase and becomes deleterious in the chronic one.

Oxidative stress is classically defined as the overproduction of reactive oxygen species (ROS) and/or decreased antioxidant defense [8] and, together with inflammation, are responsible for extensive cellular damage in the vast majority of chronic noncommunicable pathologies, such as cardiovascular [76, 77], metabolic [78], renal [79], neurodegenerative [80] diseases, cancer [81], and ageing [12].

Important, a bidirectional relationship between inflammation and oxidative stress occurs in that inflammation that arises as a defense reaction in response to ROS-mediated local tissue injury may become a source of supplementary oxyradicals. Moreover, both conditions share as a common denominator the fact that in the long run they become the major systemic pathomechanisms of the abovementioned chronic diseases [82]. The major sources of ROS are mitochondria, the NADPH oxidases, xanthine oxidase, uncoupled eNOS, and, more recently, monoamine oxidases (MAOs) [83]. The antioxidant enzymes are mainly represented by superoxide dismutases, catalase, glutathione peroxidases, thioredoxin peroxidases, and heme oxygenase-1. Any impairment of the fragile equilibrium of pro- vs. antioxidant systems is responsible for the occurrence of oxidative stress [84] that may further trigger/potentiate the inflammatory reaction. The close link between the redox status and inflammation has been systematically documented by reports on aggravated inflammatory response when either the ROS-producing enzymes were overexpressed or the antioxidant enzymes were knocked-down (reviewed in ref. [77]).

Two excellent summative reviews on the anti-inflammatory/antioxidant activity of phenylpropanoids and eugenol, respectively, were recently published [7, 85]. While the former review mainly summarized the papers reporting a decrease in the expression of various inflammatory mediators (TNF-*α*, NF-*κ*B, COX-2, IL-1𝛽, IL-4, IL-5, IL-6, iNOS, and NO) in both *in vitro* and *in vivo* models and also, of those associated with an increase in the antioxidant enzymes (superoxide dismutase, glutathione peroxidase, catalase, and glutathione peroxidase) [7], the latter addressed the effects of eugenol on the arachidonic acid- (AA-) derived mediators of inflammation. Thus, these authors reported the inhibitory effect of eugenol on prostaglandins and leukotrienes production and reduction in edema formation in several animal models of inflammation. Moreover, in human platelets, eugenol inhibited the AA and platelet-activating factor- (PAF-) induced aggregation. It has been also shown that eugenol and sodium eugenol acetate produced an inhibition in AA-induced thromboxane B_2_ and PGE_2_ formation in a concentration-dependent manner. A structurally similar compound, methyl-eugenol was evaluated in cerebral ischemic models and reported to increase superoxide dismutase and catalase activity, inhibit NO production, decrease the proinflammatory cytokines (TNF-*α*, IL-1*β*, and IL-6), and increase the anti-inflammatory ones (IL-10 and TGF-*β*), thus, indicating a potential role in the treatment of ischemia-related inflammatory diseases [85].

Leukocyte recruitment to tissue is of paramount importance in the inflammatory process. In this regard, eugenol was proven to mitigate leukocyte rolling, adhesion, and migration to the inflammatory site [86]. These results are supported by other studies performed on LPS-treated mice in which eugenol reduced lung infiltration with neutrophils/macrophages [87] and mitigated the release of inflammatory cytokines (TNF-*α*, IL-1*β*, and IL-6) [88] and the activation of NF-*κ*B [87]. Moreover, in a murine model of ovalbumin-induced allergic asthma, eugenol inhibited eosinophil lung tissue infiltration and reduced the levels of both ovalbumin-specific IgE as well as IL-4 and IL-5, the key cytokines in allergic pathologies, thereby suppressing the generation of a Th2-type immune response [89].

Recently, oral administration of eugenol in rats fed a high-fat diet (1 month) was reported to significantly decrease both total serum cholesterol and LDL cholesterol. Moreover, it mitigated inflammation and steatosis in liver sections, decreased the activities of the hepatic enzymes alanine aminotransferase and alkaline phosphatase, and increased the ones of the antioxidant enzymes superoxide dismutase and catalase in hypercholesterolemic rats. These observations further support the pleiotropic effects of the compound and delineate new avenues for research in fatty liver disease therapy [90]. Also, the anti-inflammatory and antioxidant effects of eugenol in association with cinnamaldehyde on peripheral blood mononuclear cells (PBMCs) harvested from patients with rheumatoid arthritis have been reported [91].

In recent years, the inflammatory response has been also related to the occurrence of mitochondrial dysfunction. In particular, mitochondrial DNA but also cardiolipin and N-formyl peptides are released as a result of cellular stress/damage and have been reported to induce systemic inflammation [26]. In the presence of severe inflammation, mitochondrial dysfunction was described to be associated with cell death via necrosis; conversely, in the setting of moderate inflammation, the intrinsic, mitochondrial-dependent apoptotic way of death will prevail. Interestingly, eugenol has been reported to induce early (less than 1 h exposure) mitochondrial collapse and vacuolization, followed by nonapoptotic cell death in human normal oral cells. Thus, at variance from the classic proapoptotic effect in cancer cells, eugenol might activate pyroptosis (inflammatory cell necrosis) or paraptosis (associated with mitochondria enlargement and cytoplasmic vacuolization) as cell death pathways in normal cells [92].

Therefore, targeting both chronic inflammation and oxidative stress (mainly, mitochondrial-derived) represent a promising therapeutic strategy in various pathologies. Both effects have also been described in relation to the anticarcinogenic effects of eugenol as detailed below.

### 3.2. Anticarcinogenic Activity of Eugenol

Phytochemicals are biologically active plant compounds with preventive and/or curative anticancer properties that display low toxicity and reduced side effects as compared to standard therapies. Assessing their beneficial effects as an adjunctive therapy in cancer currently represents one of the most active field of research [93]. Cancer treatment requires the inhibition of aberrant cell proliferation and destruction of malignant cells. In this respect, eugenol has been reported to elicit pro-apoptotic effects in several (but not all) tumor/cell lines.

Accordingly, a study performed in primary melanoma cell lines established from patients' tissues described an antiproliferative activity for the dimeric forms (biphenyls) of eugenol which was mild for dehydrodieugenol, higher for its *O,O*′*-*methylated form (*O,O*′-dimethyl-dehydrodieugenol), and markedly pronounced for the racemic mixture of the brominated biphenyl (*6,6*′-dibromo-dehydrodieugenol) (S7) [94].

In a murine model of skin cancer, Kaur et al. found that treatment with eugenol did not influence tumor development, but succeeded to decrease the tumor size [95]. The anticarcinogenic effect of eugenol was accompanied by anti-inflammatory properties, as shown by the reduction of several inflammatory markers such as cyclooxygenase-2 (COX-2), nitric oxide synthase (iNOS), cytokine levels (IL-6), tumor necrosis factor-alpha (TNF-*α*), and prostaglandin E2 [95]. Moreover, in a mouse skin cancer model, eugenol displayed chemopreventive properties, reducing the incidence and size of skin tumors and improving animal survival rates through apoptosis stimulation, cellular proliferation inhibition, and restriction of skin carcinogenesis at the dysplastic stage via c-Myc and H-ras oncogene downregulation and p53 tumor suppressor gene expression upregulation [96]. The tumor-suppressive effects of eugenol in skin cancers has been described to occur in relation to human melanoma and was associated with tumor size reduction, delay in tumor growth, and prevention of metastasis [97].

In a rat model of chemically-induced gastric cancer, treatment with eugenol decreased tumor incidence to 16.66%. Eugenol treatment triggered apoptosis via the mitochondrial pathway through the modulation of Bcl-2 proteins, apoptotic protease activating factor 1 (Apaf-1), caspases and cytochrome *c*, and limited angiogenesis by modifying the activity of the matrix metalloproteinases (MMP), vascular endothelial factor (VEGF), and tissue inhibitor of metalloproteinase-2 (TIMP-2) [98, 99]. Similarly, in a human lung adenocarcinoma cell line, reduction of the MMP-2 (along with phosphate-Akt) expression was demonstrated after eugenol administration, leading to inhibition of cell viability and impaired cell migration and invasion [100].

Moreover, in several human breast cancer cell lines, the epoxide forms of eugenol, lupeol, and lutein have been reported to induce apoptosis [101], while methyl-eugenol inhibited cancer cell proliferation [102]. These effects were recapitulated in the case of eugenol as well, via the downregulation of the breast cancer oncogene E2F1 and its antiapoptosis target survivin and upregulation of the cell cycle arrest-inducing protein p21^WAF1^, respectively [103]. Indeed, the proapoptotic effect of eugenol was confirmed not only in the case of breast cancer but also in human oral squamous carcinoma cells [104] and human cervix cancer and melanoma lines, respectively [105]. Treatment with this compound led to cell cycle deregulation and DNA damage via cytoplasmic membrane disruption, ROS overproduction, mitochondrial membrane potential decrease, and the downregulation of proliferating cell nuclear antigen, an essential factor in DNA replication and repair [105]. Excessive ROS generation, dissipation of the mitochondrial membrane potential, and DNA fragmentation have also been reported by other studies as mechanisms of eugenol-induced apoptosis. Thus, in a human colorectal adenocarcinoma cell line, these effects were accompanied by p53 and caspase-3 activation [106], while in a human promyelocytic leukemia cell line, the reduction of the antiapoptotic Bcl-2 protein level and the release of cytochrome *c* into the cytosol were recorded [107]. However, it must be noted that eugenol was reported to also induce nonapoptotic cell death through oxidative stress and reduction of ATP utilization in a human oral squamous cell carcinoma line [108].

The anticarcinogenic (chemopreventive-antioxidant and cytotoxic, prooxidant, and proapoptotic) effects of eugenol were the topic of a recent excellent review [109].

### 3.3. Modulation of Mitochondrial Metabolism by Eugenol

Mitochondrial dysfunction is currently accepted as the central pathomechanism of cancer. Therefore, targeting mitochondrial metabolic pathways has emerged as a valuable strategy to inhibit tumor growth. The mitochondria-targeted drugs or phytochemicals induce selectively disruption of cancerous mitochondria (and subsequent death of malignant cells) via several mechanisms, such as inhibition of respiratory function and ATP depletion, induction of the mitochondrial permeability transition, and, the previously mentioned, mitochondrial DNA damage [110].

Eugenol's effect on mitochondrial respiration can be traced back to the late 70s when Cotmore et al. firstly reported in isolated rat liver mitochondria a dose-dependent inhibition, particularly of the nicotinamide adenine dinucleotide- (NAD-) supported respiration (using glutamate as substrate) together with the uncoupling of the oxidative phosphorylation from the electron transport [111]. Several years later, Usta et al. provided further insights into the effects of the compound on mitochondrial function in the same *in vitro* model. In brief, these authors demonstrated that eugenol dose-dependently elicited the (i) inhibition of NADH oxidase (complex I of the electron transport system), (ii) reduction of mitochondrial membrane potential (*ΔΨ*m), and (iii) stimulation of the ATPase activity of F1F0-ATP-ase (complex V) with subsequent ATP depletion in rat liver mitochondria [112].

Eugenol is a weak lipophilic acid (i.e., it might permeate the mitochondrial membranes and release a H^+^ into the matrix) and also an analogue of dinitrophenol (a classical mitochondrial uncoupler). Together with complex I inhibition, these properties might be responsible for the dissipation of the proton gradient across the inner membrane (normally used by the ATP synthase to generate ATP); subsequently, the enzyme will work in the reverse mode and become an energy-dissipating structure [113]. Of note, eugenol had no effect on succinate dehydrogenase (complex II of the electron transport system) activity in isolated rat mitochondria. More recently, the same group reported the chemosensitivity of a human breast cancer cell line MCF-7 to eugenol. The compound elicited a dose-dependent: (i) decrease in cellular viability and proliferation (EC_50_: 0.9 mM), (ii) decrease in ATP level and mitochondrial membrane potential, (iii) increase in reactive oxygen species generation, (iv) release of cytochrome-c and lactate dehydrogenase, and (v) nonapoptotic Bcl-2 independent toxicity [114]. The last finding is in line with the results from the group of Sakagami, which confirmed the nonapoptotic cell death in three human normal oral cell types (gingival fibroblast, periodontal ligament fibroblast, and pulp cell) yet with no effect on ATP utilization (except for periodontal fibroblasts). Importantly, Sakagami et al. also reported that eugenol (2 mM) elicited a rapid suppression (after 20 min incubation) of the tricarboxylic acids cycle in all three cell lines mentioned above, whereas the intracellular concentration of glycolytic metabolites slightly increased [92]. In an elegant study, Yan et al. further provided mechanistic insights into the signal transduction underlying the anticarcinogenic effect of eugenol in MCF10A human breast epithelial cells transfected with the H-ras oncogene (MCF10A-ras). These authors reported that eugenol (200 *μ*M) suppressed cell growth and inhibited oxidative phosphorylation and fatty acids oxidation via the downregulation of the c-Myc/PGC-1*β*/ERR*α* pathway. Of note, the latter was upregulated in the breast cancer MCF10A-ras cells but not in the untransformed MCF10A cells [115].

In line with these observations, we aimed to investigate the effect of eugenol, free, or encapsulated in polyurethane nanoformulations [25], on both mitochondrial bioenergetics and glycolysis in SCC-4 human squamous cell carcinoma cells by means of the extracellular flux analyzer Seahorse XF24e (Agilent Technologies Inc.). This automatic platform provides a simultaneous measurement of oxygen consumption rate (OCR) as an indicator of mitochondrial respiration, and the extracellular acidification rate (ECAR) as an indirect measurement of anaerobic glycolysis, according to a previously described method [116]. In brief, cellular metabolic activity was challenged with the classic modulators of the mitochondrial electron transport chain: the first automatic injection was performed using oligomycin (1 *μ*g/ml), the inhibitor of the mitochondrial ATP synthase; FCCP (3 *μ*M), a classic uncoupler, was further injected, followed by antimycin A (5 *μ*M), the inhibitor of mitochondrial complex III. OCR was reported in units of pmoles/min and ECAR in mpH/min. SCC-4 human squamous carcinoma cells were incubated for 24 h with eugenol (free or incorporated in polyurethane structures), and we found that free eugenol (50 *μ*M) induced a decrease of OCR parameters (i.e., inhibition of mitochondrial respiration) coupled with an increase of ECAR (i.e., stimulation of glycolysis); surprisingly, opposite effects were recorded for eugenol nanoformulations, i.e., an increase in basal and maximal respiration (OCR) plus a decrease in glycolysis (ECAR) [116]. The effects of free eugenol are in line with the abovepresented literature data, yet the paradoxical effect of nanostructures requires further investigations.

**Figure 1:**
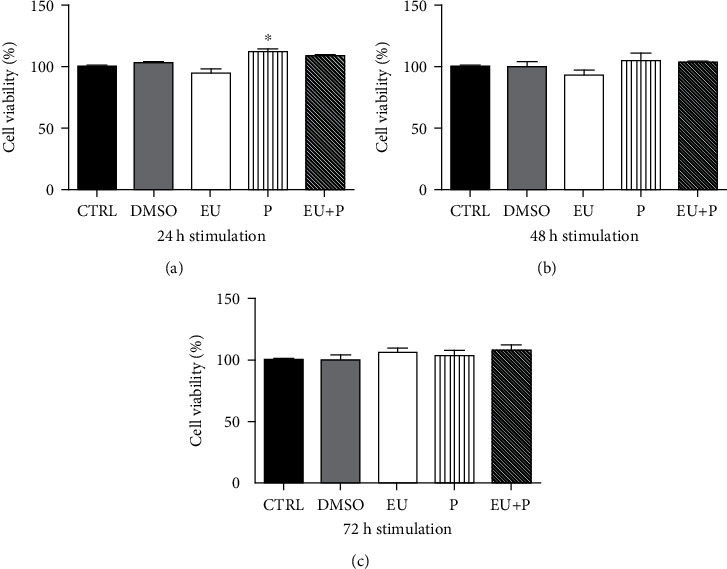
The time-dependency of HaCaT cell viability. (EU: free eugenol; P: polyurethane nanostructure alone; EU + P: eugenol included as nanoformulation). Data are presented as mean ± SD. Experiments were performed in triplicate (^∗^*p* < 0.05 vs. Ctrl).

We further recapitulated the experiments using the normal HaCaT human keratinocytes incubated with 50 *μ*M free eugenol (EU), polyurethane particles alone (P), and EU encapsulated in polyurethane structures (EU + P) for 24, 48, and 72 h. First, the cytotoxic effect of the compounds on HaCaT cells was assessed at 24 h—Figure 1(a), 48 h—Figure 1(b), and 72 h—Figure 1(c), respectively. Cytotoxicity was evaluated by means of the MTT assay, as previously described [117]. HaCaT cells were seeded in 96-well culture plates (1 × 10^4^ cells/well) and allowed to attach. Next, the medium was replaced and cells were incubated for 24, 48, and 72 h, respectively, with the tested compounds. Cells were randomized into 5 groups: control group—untreated cells (CTRL); DMSO group—cells treated with 50 *μ*M DMSO used to prepare the free EU stock solution (DMSO); group treated with 50 *μ*M free EU (EU); group treated with 50 *μ*M polyurethane structures which were used to encapsulate EU (P); and group treated with 50 *μ*M encapsulated EU (EU + P). A volume of 10 *μ*L of 5 mg/mL MTT solution from the MTT toxicology assay kit (Sigma-Aldrich) was added in each well. In the presence of NADPH-dependent cellular oxidoreductases, MTT precipitated as the insoluble formazan (during 4 h). The reduced MTT was measured spectrophotometrically at 570 nm, using a microplate reader (xMarkMicroplate Spectrophotometer, Bio-Rad). All experiments were performed in triplicate.

At 24 h poststimulation, EU elicited a discrete, nonsignificant cytotoxic effect, whereas the polyurethane structures (P, EU + P) provoked an unexpected mild increase in cell viability (Figure 1(a)). Similar results were obtained at 48 h of stimulation (Figure 1(b)), while at 72 h, the EU group also showed a trend for an increase in cell viability vs. CTRL (Figure 1(c)).

**Figure 2:**
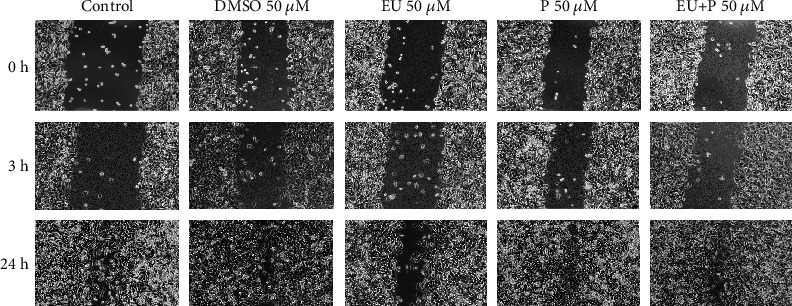
The effects of compounds (50 *μ*M) on HaCaT cell migration. (EU: free eugenol, P: polyurethane nanostructure alone, EU + P: eugenol included as nanoformulation). Pictures were taken at 0, 3, and 24 h poststimulation (10× magnification).

We further evaluated the effect of the compound (50 *μ*M) on HaCaT cell migration using the scratch assay, as previously described [118]. To this aim, 2 × 10^5^ cells/well were cultured in 12-well plates for 48 h prior to the experiment. Scratches were drawn in well-defined zones of the cells monolayer (at a confluence of 90%) using a sterile pipette tip. The detached cells were removed by washing with PBS before stimulation, and afterward, the cells were incubated with the compounds. Images of the cells were taken at the starting point of the experiment, and after 3 and 24 h, respectively, using the inverted microscope Olympus IX73 and the cellSense Dimension software.  Figure 2 shows that P and EU + P groups exhibited a promigratory effect upon HaCaT cells at 24 h poststimulation vs. the control group. EU alone elicited at 24 h stimulation a mild reduction of cellular migration as compared to P/EU + P groups.

**Figure 3:**
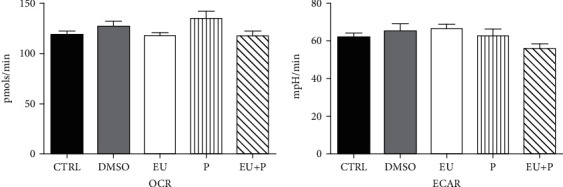
The effects of 24 h incubation of HaCaT cells on OCR and ECAR. Data are presented as mean ± SD. Experiments were performed in triplicate.

**Figure 4:**
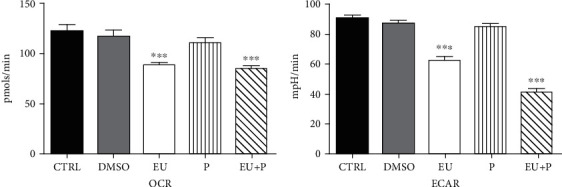
The effects of 48 h incubation of HaCaT cells on OCR and ECAR. Data are presented as mean ± SD. Experiments were performed in triplicate (^∗∗∗^*p* < 0.001 vs. Ctrl).

**Figure 5:**
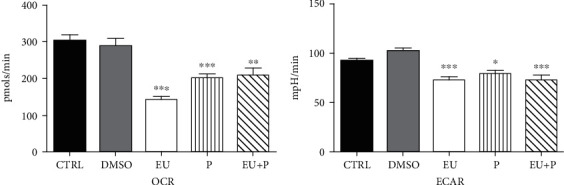
The effects of 72 h incubation of HaCaT cells on OCR and ECAR. Data are presented as mean ± SD. Experiments were performed in triplicate (^∗^*p* < 0.05; ^∗∗^*p* < 0.01; ^∗∗∗^*p* < 0.001 vs. Ctrl).

Finally, we evaluated the bioenergetic profile of HaCaT human keratinocytes treated with the compounds (50 *μ*M) at 24 h—Figure 3, 48 h—Figure 4, and 72 h—Figure 5 using the Seahorse extracellular flux analyzer, as previously described.

No significant effect on OCR and ECAR was observed after 24 h of treatment (Figure 3). At variance, a significant metabolic inhibition was noticed for the two other periods of exposure (Figures 4 and 5). Thus, in the EU and EU + P groups, both OCR and ECAR significantly decreased at 48 h of incubation (Figure 4). Surprisingly, at 72 h, not only EU and EU + P but also P, the polyurethane particles *per se*, induced a significant inhibitory effect on both metabolic pathways, oxidative phosphorylation, and glycolysis (Figure 5).

The polyurethane nanostructures represent a safe formulation as previously reported [25], since they neither elicited a cytotoxic effect on HaCaT cells regardless of the incubation period (Figures 1(a)–1(c), P group) nor interfered with their migration (Figure 2, P group). However, the nanoformulations significantly depressed the cellular metabolism at 72 h (Figure 5, P group).

## 4. Conclusions

Eugenol is a versatile molecule that has successfully survived the test of time in dental medicine. Nowadays, it has emerged as a promising phytochemical in the armamentarium of adjunctive anticancer therapeutics via the modulation of chronic inflammation, oxidative stress, and mitochondrial dysfunction, the major pathomechanisms of noncommunicable diseases. The current understanding of the signaling pathways responsible for eugenol interaction with cellular metabolism is far from being elucidated. Further studies aimed at characterizing its effects on bioenergetics and mitochondrial metabolism in both normal and malignant cell lines are fully warranted.

## Figures and Tables

**Table 1:** The “good” vs. the “bad” side of eugenol and ZOE-based materials in dentistry.

Type of material	Type of study	Beneficial effects	Deleterious effects	Ref.
Eugenol	*In vivo*	(i) Anti-inflammatory properties (ii) Antinociceptive activity	—	[33]
Eugenol	*In vitro*	(i) Antimicrobial activity against the periodontal pathogens	—	[35]
Eugenol	*In vitro*	(i) Antibacterial activity against oral pathogens (ii) Cario-protective action (iii) Antifungal activity (iv) Cytotoxic action against several cancer cells (v) Antimutagenic action	—	[36]
Eugenol	*In vitro*	—	(i) Suppresses polymerization (ii) Reduces the mechanical properties of composite resins but to a distance of less than 100 nm	[29]
Eugenol	*In vivo*	(i) Promoted wound healing (ii) Anti-inflammatory action (iii) Analgesic action	—	[44]
Eugenol	*In vitro*	(i) No DNA strand break activity	(i) Cytotoxic effects to oral mucosal fibroblasts (ii) Decrease of cellular ATP level (iii) Inhibition of lipid peroxidation	[53]
Eugenol	*In vitro*	(i) Concentration-dependent effect on cellular growth	(i) Decreased cell survival (ii) Decreased collagen synthesis	[55]
Eugenol	*In vitro*	(i) Apoptosis of oral SCC cells line	(i) Low tumor-specificity	[56]
Eugenol	*In vitro*		(i) Toxic effects on dental pulp fibroblasts (even at very low concentrations)	[60]
Eugenol	*In vivo*		(i) Hypersensitivity response of oral mucosa (ii) Cytotoxic effects	[63]
Eugenol	*In vitro*		(i) Retardation of the resin dental materials polymerization	[67]
ZOE	*In vitro*	(i) Anti-inflammatory effect (ii) Immunomodulatory effects	(i) Decrease in cell viability (ii) Cytotoxic effect in high concentrations	[32]
ZOE	*In vivo*	(i) Anaesthetic action (ii) Inhibition of intradental nerve activity	—	[43]
ZOE	*In vitro*	(i) Good mechanical properties as a base under composite materials	—	[71]
ZOE	*In vivo* *In vitro*	(i) Anti-inflammatory effects (ii) Inhibition of synthesis of cyclooxygenase derivatives	—	[39]
ZOE	*In vitro*	—	(i) Increased cytotoxicity and apoptosis of human periodontal ligament fibroblasts	[46]
ZOE	*In vitro*	—	(i) High cytotoxicity for fibroblasts cell lines	[47]
ZOE	*In vitro*	—	(i) Cytotoxic activity (ii) Inhibition of the metabolic activity	[48]
ZOE	*In vitro*	—	(i) High cytotoxicity on human periodontal ligament cells and V79 cells	[49]
ZOE	*In vitro*	—	(i) Cytotoxic activity on human periodontal ligament fibroblasts and L929 cells	[50]
ZOE	*In vitro*	—	(i) Negative effects on microtensile bond strength of adhesives to dentin	[59]
ZOE (Endomethasone)	*In vitro*	—	(i) Decrease in bond strength to the root dentin	[68]
ZOE (Endomethasone)	*In vivo*	—	(i) Periapical inflammation with granulomatous reaction around the sealer particles	[51]
ZOE + hydrocortisone (Endomethasone N)	*In vitro*	(i) Decreased cell migration and secretion of IL-6 and TNF-*α* by human periodontal ligament cells	—	[62]

**Table 2:** Overview of the anti-inflammatory, antioxidant, and anticarcinogenic effects of eugenol.

Eugenol properties	Parameters/tumor type	Biological effects	Ref.
Anti-inflammatory	Histological quantification of liver inflammatory foci/microscopic field	Decrease of the liver inflammatory cell infiltration	[90]

Anti-inflammatory	Cytokine levels	Decrease of the TNF-*α* and IL-6 level in the culture supernatant of RA-PBMCs	[91]

Anti-inflammatory	Mouse skin expression of COX-2 cytokine levels	Decrease of skin COX-2 expression and serum TNF-*α*, IL-6, and PGE2 level in TPA-treated mice	[95]

Anti-inflammatory	Leukocyte migration	Decrease of the number and adherence of leukocytes	[86]

Anti-inflammatory	Cytokine levels	Inhibition of lung infiltration with eosinophils decrease of IL-4 and IL-5 levels	[89]

Anti-inflammatory	Cytokine levels	Inhibition of TNF-*α*, IL-1*β*, and IL-6 release	[88]

Anti-inflammatory	Inflammatory cells cytokine level NF-*κ*B activation	Inhibition of lung infiltration with neutrophils/macrophages; reduction of TNF-*α* release and of NF-*κ*B activation	[76]

Anti-inflammatory	Inflammation-related gene expression (NF-*κ*B, IL-1*β*, and TNF-*α*)	Inhibition of NF-*κ*B and TNF-*α* gene expression	[61]

Antioxidant	Antioxidant enzyme (SOD and CAT) activity	Increase of serum SOD and CAT activity	[90]

Antioxidant	Intracellular ROS production and reduced glutathione level antioxidant enzyme (SOD, CAT, and GPx) activity	Decrease of ROS generation and increase of reduced glutathione level in RA-PBMC increase of SOD, CAT, and GPx activity in RA-PBMC culture	[91]

Antioxidant	Cutaneous glutathione level and glutathione reductase, CAT, and GPx activity	Increase of cutaneous glutathione level and glutathione reductase, CAT, and GPx activity in TPA-treated mice	[95]

Anticarcinogenic	MCF-7 human breast cancer cells	Inhibition of human breast cancer cell proliferation	[102]

Anticarcinogenic	Mouse skin cancer	Reduction in tumor size and incidence	[95]

Anticarcinogenic	Mouse skin cancer	Restriction of skin carcinogenesis at the dysplastic stage	[96]

Anticarcinogenic	Rat gastric cancer	Inhibition of gastric carcinoma development through NF-*κ*B suppression	[99]
		Apoptosis stimulation through modulation of Bcl-2 proteins, Apaf-1, caspases, and cytochrome c inhibition of invasion and angiogenesis by MMP activity and VEGF and TIMP-2 expression modulation	[98]

Anticarcinogenic	HSC-2 human oral squamous cell carcinoma cell line	Nonapoptotic cell death through oxidative stress and reduction of ATP utilization	[108]

Anticarcinogenic	Human melanoma cells B16 xenograft mouse model	Tumor size reduction and delay in tumor growth; prevention of metastasis	[97]

Anticarcinogenic	Human breast cancer cells	Proliferation inhibition and apoptosis stimulation through down-regulation of survivin and the E2F1 transcription factor	[103]

Anticarcinogenic	A549 human lung adenocarcinoma cells	Inhibition of cell proliferation, migration, and invasion through modulation of MMP activity and the PI3K/Akt pathway	[100]

Anticarcinogenic	HCT-15 and HT-29 human colorectal adenocarcinoma cells	Apoptosis stimulation through the reduction of *ΔΨ*m with oxidative stress and DNA fragmentation	[106]

Anticarcinogenic	HL-60 human promyelocytic leukemia cells	Apoptosis stimulation through oxidative stress, MPT, and cytochrome c release, reduction of Bcl-2 level and DNA fragmentation	[107]

Anticarcinogenic	Human KB oral squamous carcinoma cells and DU-145 androgen-insensitive prostate cancer cells	Cell growth inhibition and apoptosis stimulation	[104]

Anticarcinogenic	MDA-MB-231, MCF-7 (breast cancer lines), SIHA (cervix cancer lines), SK-Mel-28, and A2058 (melanoma lines)	Apoptosis stimulation through cell cycle deregulation and DNA damage, ROS overproduction, disruption of the cytoplasmic membrane, mitochondrial failure, PCNA downregulation	[105]

RA-PBMCs: PBMCs isolated from rheumatoid arthritis patients; TPA: 12-*O*-tetradecanoylphorbol-13-acetate; MAPK: mitogen-activated protein kinases; SOD: superoxide dismutase; CAT: catalase; GPx: glutathione peroxidase; PARP: polyadenosinediphosphate-ribose polymerase; MPT: mitochondrial permeability transition; PCNA: proliferation cell nuclear antigen; *ΔΨ*m: mitochondrial membrane potential.
